# Adenoids in Infancy

**Published:** 1902-02-22

**Authors:** 


### ADENOIDS IN INFANCY.

We are all accustomed to recognise the presence of adenoid growths in the naso-pharynx as the cause of many symptoms in children, and to see these evil consequences vanish when their cause has been removed. Perhaps we are not so much in the habit of looking out for these injurious growths in early infancy. According to Dr. Hutchison,[1] there is reason to believe that adenoids may be a congenital abnormality. In a clinical lecture recently delivered at the Ormond Street Hospital for Sick Children he mentioned a series of symptoms which, in his experience, had been associated with and apparently caused by the presence of these growths. A child, aged five months, was brought with a history that it had been snuffling at the nose, and had had difficulty in breathing ever since it was born. There was no suspicion of syphilis. The respiration was laboured, and the lower interspaces were drawn in during inspiration. Quite a mass of fibrous adenoids was found, and on their removal the child was brought back with almost complete relief from all its symptoms. Another group of cases shows as their chief symptom cough. A child only two months old was brought on account of choking and paroxysmal cough, the paroxysms being accompanied by the passing of fæces. The lungs were healthy, but the naso-pharynx was stuffed with adenoids. Another baby had snoring inspiration, and was only able to sleep in snatches, out of which it started up in partial asphyxia. During the day it had paroxysms of choking cough, and sweated profusely about its head. Great and immediate relief followed operation. Another class of case exhibits stridulous respiration as the chief characteristic. Difficulty of swallowing is the complaint in another set. This may arise quite early, very soon after birth, and may render the child unable to take the bottle. In some of the latter cases the difficulty is clearly due to interference with the respiration. The infant has only its mouth to both breathe and suck by; and consequently, when it is using its mouth for sucking, it cannot be using it for breathing. It thus gets short of breath, and thereupon instantly ceases to suck. But even feeding with a spoon may be difficult, and Dr. Hutchison thinks that in these cases the adenoids may interfere mechanically with the act of deglutition, perhaps by preventing that elevation of the soft palate into the naso-pharynx which is a normal occurrence during healthy deglutition. Before turning to the consideration of other subjects Dr. Hutchison pointed out that in diagnosing the condition one has to go much by symptoms, and that therefore the association of the symptoms mentioned with adenoids must be borne in mind. One might think, he said, that when one suspects adenoids a local examination ought to settle the matter at once. But, unfortunately, it is difficult to examine the naso-pharynx of these small children satisfactorily. There is an extremely small space between the soft palate and the posterior wall of the pharynx in a young infant, so that one can hardly get one's finger up behind the nose.

[1] Clinical Jour., Jan. 29.

